# Impact of a computer-assisted decision support system (CDSS) on nutrition management in critically ill hematology patients: the NUTCHOCO study (nutritional care in hematology oncologic patients and critical outcome)

**DOI:** 10.1186/s13613-019-0527-6

**Published:** 2019-05-07

**Authors:** Florence Ettori, Aurélia Henin, Christophe Zemmour, Laurent Chow-Chine, Antoine Sannini, Magali Bisbal, Frédéric Gonzalez, Luca Servan, Jean Manuel de Guibert, Marion Faucher, Jean Marie Boher, Djamel Mokart

**Affiliations:** 1 Intensive Care Unit, Department of Anesthesiology and Critical Care, Institut Paoli Calmettes, 232 Boulevard de Sainte Marguerite, 13009 Marseille Cedex 09, France; 2 Unité de Biostatistique et de Méthodologie, Institut Paoli-Calmettes, Marseille, France; 3 INSERM, IRD, SESSTIM, Aix Marseille Université, Marseille, France

## Abstract

**Background:**

Mortality of critically ill hematology (HM) patients has improved over time. Thus, those patients require an extensive diagnostic workup and the optimal use of available treatments. There are no data regarding nutrition strategy for critically ill HM patients, while nutritional support is crucial for both HM and critically ill patients. We hypothesized that the implementation of a computer-assisted decision support system (CDSS), designed to supervise a nutritional intervention by a multidisciplinary team, would be able to increase guidelines adherence and outcomes.

**Results:**

In this before/after study, 275 critically ill hematology patients admitted to the ICU over 5-year period were included. Energy and protein intakes were delivered using standard protocol in the 147 patients (53%) of the ‘before group’ and using a CDSS in order to reach every day predefined caloric and protein targets accordingly to the catabolic or anabolic status in the 128 patients (47%) of the ‘after group.’ Using a Poisson regression, we showed that the use of CDSS allowed to reach a relative increase in the rate of days in compliance with caloric (1.57; 95% confidence interval (CI), [1.17–2.10], *p* = 0.0025) and protein targets (3.86 [2.21–6.73], *p* < 0.0001) in the ‘after group’ by more than 50% as compared with the ‘before group.’ Interestingly, compliance rates were low and only reached 30% after intervention. Hospital mortality, ICU-acquired infection, and hospital, and ICU length of stay were similar in the two groups of patients. Importantly, exploratory analysis showed that hospital mortality was lower in the ‘after group’ for neutropenic and severely ill patients.

**Conclusion:**

For critically ill hematology patients, the use of a nutritional CDSS allowed to increase the days in compliance with caloric and protein targets as compared with no CDSS use. In this context, overall hospital mortality was not affected.

**Electronic supplementary material:**

The online version of this article (10.1186/s13613-019-0527-6) contains supplementary material, which is available to authorized users.

## Introduction

HM patients increasingly require admission to the intensive care unit (ICU) for life-threatening events related to the malignancy and/or treatments [1, 2]. The prognosis of HM patients has improved considerably over time, in fact mortality in HM patients septic shock has fallen by 30% [3], while in patients receiving invasive mechanical ventilation (IMV) improvement has resulted in a sharp drop in mortality, from nearly 90% to about 40% [4]. Thus, critically ill HM patients require an extensive diagnostic workup [5] and the optimal use of available treatments [6]. Nutritional support is crucial in critically ill patients. Guidelines recommend early enteral feeding supplying 25 kcal/kg per day during the acute phase of critical illness [7, 8] with however a low level of evidence. For HM patients treated with myeloablative conditioning for allogeneic stem cell transplantation, early nutritional intervention has proven a benefit on outcome and mortality outside the ICU [9]. There are no data regarding nutrition strategy for HM patients during ICU stay. As most of ICU therapies, nutritional support has become more complex requiring tight supervision and monitoring. Thus, computer-assisted decision support systems (CDSSs), designed to implement rational care strategies based on guidelines and pathways, have been developed and studied over the past several years [10]. For this study, we hypothesized that implementation of a CDSS, designed to actively supervise a nutritional intervention by a multidisciplinary team, would be able to increase guidelines adherence and outcomes.

## Methods

### Study periods

This retrospective ‘before/after’ cohort study was conducted over two time periods within 5 years totaling 3501 patient days in the ICU for 275 patients. The pre-interventional period (‘before period’) took place from November 2009 to March 2011, the post-interventional period (‘after period’) from November 2011 to November 2014. We compared two chronological cohorts: the ‘before group’ with unmonitored nutritional care and the ‘after group’ with updated nutritional care (computer assisted, guideline updated, immunonutrient supplemented). Patients and most of clinico-biological data were retrospectively identified and analyzed using our prospective ICU database included in our ICU information system (MetaVision^®^, iMDsoft, Tel Aviv, Israel). Following the ‘before period,’ a CDSS module was locally designed and added to our ICU information system as a tool to provide clinicians with essential guideline-based information concerning nutritional support in the ICU and hematology patients [7, 11–13]. Details concerning the implementation of the nutrition protocols during the 2 periods are provided in the Additional file 1. The study was approved by the Paoli-Calmettes Institute institutional review board which waived the need for informed consent.

### Aims of the study

By the use of CDDS, our primary objective was to demonstrate a significant increase in the number of days in compliance with calories target, defined as days on which caloric delivery fitted the target of 25 kcal/kg/day ± 15% during the aggression period, or 35 kcal/kg/day ± 15% during stable recovering periods, and in the number of days in compliance with protein target, defined as days on which protein delivery fitted a target of 1.5 g/kg/day ± 15%. Secondary clinical objectives were to assess the impact of the use of the CDSS on morbidity (ICU and hospital lengths of stay, duration of IMV, antimicrobial, renal replacement, and vasopressors treatments) and hospital survival for the overall patients, exploratory analysis was also realized for hematopoietic stem cell transplant (HSCT) recipients, neutropenic and severe patients defined as SAPS II > 56 on ICU admission [14–16]. Nutritional secondary objectives were to assess the impact of the use of the CDSS on cumulative days of enteral, parenteral, and total caloric and protein cumulative balance on day 3, day 5, and day of ICU discharge.

### Targets and nutrition protocols

Protocols were based on ESPEN guidelines [7, 11–13] that were locally adapted and validated by the ICU staff. During the study periods, the energy target recommendation was 25 kcal/kg/day and 35 kcal/kg/day during acute illness and recovery period, respectively. Patients were categorized as in acute phase or recovery phase at the clinician’s opinion and corresponded to any of the following situations: ongoing vasopressor treatment, renal replacement therapy (RRT), IMV, sepsis, shock, etc. Recovery period was defined as stable clinical state which could include prolonged (> 48 h) stable ventilation periods. Ideal body weight was used for calculations in case of BMI > 30 kg/m^2^ [17]. Protein recommendations were set at 1.5 g/kg/day. Enteral nutrition (EN) was used whenever possible, combined feeding when EN did not cover the prescribed target, and parenteral nutrition (PN) when EN was not feasible. Compliance with guidelines was evaluated for each patient each day of the ICU stay according to the recommended energy and protein targets. Intravenous energy was received from PN, as well as from non-nutritional energy sources (calculated by the CDSS which considered glucose, gluco-saline, or drug infusion, and fat from propofol). Daily protein delivery was also retrieved from the CDSS on days with artificial nutrition. Feeding routes were defined as PN, EN, combined (EN and PN), and oral. Since our CDSS was designed to only assist the EN and PN prescriptions, oral intake was not taken into account for the primary endpoints of the overall population. In addition, oral alimentation is commonly difficult to realize in severe hematology patients (ARF, septic shock, mucositis, HSCT recipient), thus we assume the oral intakes to be negligible in those situations.

Feeding products are described in Additional file 2.

### ICU management

Our medical institution is a tertiary care reference center for cancer patients. Patients admitted to the ICU benefit from a daily close collaboration between their referring hematologists and ICU-appointed intensivists. All consecutive patients suffering from a hematologic neoplasia were included at 48 h of ICU. Pregnant women, minors, patients admitted for, or switched within the first 48 h to palliative ICU care, patients discharged alive within the first 48 h were not included. Reasons for ICU admission were recorded based on the main symptoms at ICU admission. Acute respiratory failure was defined as oxygen saturation less than 90% or PaO_2_ less than 60 mmHg on room air combined with severe dyspnoea at rest with an inability to speak in sentences or a respiratory rate greater than 30 breaths per minute or clinical signs of respiratory distress [5]. Shock was defined as previously reported [16]. Mild malnutrition was defined as total weight loss > 5% and ≤ 10% usual body weight over the last 6 months and severe malnutrition as weight loss > 10%. Life-supporting interventions, anti-infectious agents, prophylactic treatments, urate oxidase use, and diagnostic procedures were administered at the discretion of the attending intensivists, who followed best clinical practice and guidelines. Chemotherapy, corticosteroids, hematopoietic growth factors, immunosuppressive drugs, and other cancer-related treatments were prescribed by the hematologist in charge of each patient in accordance with institutional guidelines. Etiologic diagnoses were made by consensus by the intensivists, hematologists, and consultants, according to recent definitions [1].

### Clinical and biological data

All clinical and biological data were prospectively collected, except for caloric and protein balances in the ‘before group’; however, for this group, intakes were prospectively computed by our medical system information. The following clinical data were collected during the ICU stay (Table 1), among them: age and gender; height; weight; calorie and protein intakes; caloric and protein deficit at day 3, day 5, and ICU discharge; the number of days in compliance with caloric and protein targets during ICU stay; loss of weight at ICU discharge; characteristics of the malignancy; neutropenia (absolute neutrophil count of < 0.5 × 10^9^ l^−1^); characteristics of sepsis; chronic health status as evaluated using the Knaus scale [18]; severity-of-illness scores using Simplified Acute Physiology Score II (SAPS II) [19] at admission, cause of ICU admission; therapeutic interventions in ICU, including vasopressor use, need, and duration of IMV, RRT, antimicrobial treatment; ICU and hospital length of stay; and hospital mortality. Organ failures as defined as a SOFA score of ≥ 3 for any system [20].

**Table 1:** Characteristics of the population

	Before group (*n* = 147)	After group (*n* = 128)	*p* value
Sex (female)	63 (43)	48 (37)	0.37
Age (years)	58 [46–66]	60 [52–68]	0.14
Height (cm)	170 [164–175]	170 [165–175]	0.33
Weight at ICU admission (kg)	75 [63–83]	73 [65–87]	0.32
Weight at ICU discharge (kg)	70.00 [59–80]	70.00 [60–82]	0.84
Weight loss at ICU discharge (kg)	1.00 [0.00–3.00]	0.00 [–0.50–2.00]	0.53
BMI	24 (21–28)	24 (22–27)	0.84
Nutritional status			
No or Mild malnutrition	119 (81)	95 (74)	0.18
Severe malnutrition	27 (19)	33 (26)	0.14
Oral alimentation at ICU discharge	92 (63)	75 (59)	0.50
SAPSII score	46 [36–59]	48.50 [40–58]	0.38
Severe patients (SAPSII > 56)	41 (28)	40 (31)	0.54
Comorbidities			
Cardiovascular	46 (31)	47 (37)	0.34
Respiratory	37 (25)	42 (33)	0.73
Hepatic	8 (5)	5 (4)	0.55
Renal	11 (7)	5 (4)	0.21
Neurologic	11 (7)	8 (6)	0.69
Diabetes	16 (11)	12 (9)	0.70
Hematology disease			
AML	56 (38)	35 (27)	0.06
ALL	18 (12)	4 (3)	0.006
Lymphoma	40 (27)	50 (39)	0.04
Myeloma	17 (12)	14 (11)	0.87
Autologous SCT	27 (18)	30 (23)	0.30
Allogeneic SCT	32 (22)	38 (30)	0.13
Neutropenia	43 (29)	57 (45)	0.009
CML, CLL	16 (11)	25 (20)	0.04
Complete remission	43 (34)	31 (24)	0.1
Main cause for ICU admission			
ARF	50 (34)	56 (44)	0.1
Shock	48 (33)	35 (27)	0.36
AKI	18 (12)	6 (5)	0.03
Neurologic failure	11 (7)	15 (12)	0.23
Others	20 (14)	16 (12)	0.79
Organ support			
IMV	72 (49)	59 (46)	0.72
Vasopressors	83 (56)	72 (56)	1
RRT	32 (22)	35 (27)	0.32
ICU length of stay (days)	11 (13)	14 (13)	0.06
Hospital mortality	72 (49)	54 (42)	0.28
Hospital length of stay (days)	38 (41)	37 (28)	0.94

*AKI* acute kidney injury, *ALL* acute lymphoblastic leukemia, *AML* acute myeloid leukemia, *ARF* acute respiratory failure, *BMI* body mass index, *CLL* chronic lymphocytic leukemia, *CML* chronic myelogenous leukemia, *HSCT* hematopoietic stem cell transplantation, *ICU* intensive care unit, *IMV* invasive mechanical ventilation, *RRT* renal replacement therapy, *SAPSII* Simplified acute physiology score

### Statistical analysis

Individual data collected prior to ICU were summarized using counts (frequencies) for qualitative variables and medians [25th–75th percentiles] or means [standard deviations (SD)] for quantitative variables. The primary objective was to compare the number of days in compliance with calories target between the study cohorts enrolled prior and after initiation of CDSS intervention, respectively. Analysis of the primary endpoint was performed using Poisson regression including terms for cohort (after vs. prior CDSS) and length of ICU stay as an offset term to account for individual duration of exposure. A multivariate regression was then performed to confirm the results, by adding as covariates neutropenic, severe and allogeneic SCT statuses. Estimate of intensity rate ratio (IRR) derived from Poisson regression was used to assess the difference in the number of days in compliance between the two cohorts. Correction for over dispersion in variance estimation was used to derive robust 95% confidence intervals for IRR. Similar analyses were performed for the number of protein compliant days, treatment days IMV, vasopressors, RRT, and antibiotics. Exploratory similar analyses of the number of caloric and protein compliant days were also performed in neutropenic, severe and allogeneic HSCT patients, respectively; in order to account for multiple comparisons, a Bonferroni correction was applied to these analyses and the level of significance was set to 0.05/6 = 0.008. In each cohort, cumulative incidences of death during ICU stay were estimated using the Prentice’s method by considering patient alive at ICU discharge as a competing risk and compared using Gray’s tests. Other secondary endpoints were compared across the two groups of patients by using Chi-square or Fisher’s exact tests for qualitative variables, and Wilcoxon rank-sum tests for quantitative variables.

A total of 252 patients was initially planned in this study (126 in each cohort). This sample size was determined using Cook’s formula to a detect a 50% increase in the primary endpoint using CDSS (IRR = 1.5) with 95% power and 5% error risk, assuming equal period of accrual in each cohort; an expected length of stay in ICU of 7 days, an intensity rate of 0.40 in the first cohort and a default value for over dispersion parameter (*ϕ* = 1).

All statistical analyses were performed using the Statistical Analysis System (SAS), version 9.3 (SAS Institute Inc, Cary, NC, USA).

## Results

### Clinical parameters (Table 1)

Two hundred seventy-five patients were included in the present study, 147 (53%) in the ‘before group’ and 128 (47%) in the ‘after group.’ Clinical characteristics of patients are presented in Table 1. Briefly, age was 59 years [49–67] and SAPSII score was 47 [37–59]. Main comorbidities were cardiovascular disease for 34% (*n* = 93) of subjects, respiratory 29% (*n* = 79), hepatic 5% (*n* = 13), renal 6% (*n* = 16), neurological 7% (*n* = 19), and diabetes mellitus 10% (*n* = 28). Regarding hematological malignancy, 113 (41%) patients presented with acute leukemia, 90 (33%) with lymphoma and 41 (15%) with chronic leukemia. At ICU admission, 100 (36%) patients were neutropenic, 70 (25%) and 57 (21%) patients benefited from allogeneic and autologous HSCT, respectively. One hundred six (38%) patients presented with ARF and 83 (30%) with shock. During ICU stay, 131 (48%) patients required IMV, 155 (56%) vasopressors and 67 (24%) RRT. Hospital mortality was 46% (*n* = 126).

### Nutritional parameters

Regarding energy delivery (Table 2), during the ICU stay the rate of days in compliance with caloric target had improved in the ‘after group’ by a 10% absolute increase and 50% relative increase in percentage of energy target reached as compared with the ‘before group’ (28.8% [24.6–33.7%] vs. 18.4% [14.4–23.5%]; IRR = 1.57; 95% confidence interval (CI), [1.17–2.10], *p* = 0.0025). This effect was confirmed in multivariate analysis (28.3% [24.0–33.5%] vs. 20.0% [15.6–25.7%], IRR = 1.42 [1.05–1.94], *p* = 0.02). We did not observe this trend in neutropenic, severe, or HSCT patients (Table 2). In accordance, daily calories intake was significantly higher in the ‘after group’ as compared with the ‘before group’ (Additional file 3: Table S1). Between the two periods, the rate of days with PN significantly increased (IRR = 1.29 [1.10–1.52], *p* = 0.0021), while a non-significant trend was described for EN (IRR = 1.90 [0.98–3.66], *p* = 0.056) as well as for combined nutrition (EN + PN) (Additional file 4: Table S2). A specific nutritional focus on day 3 established that only three (2%) patients benefited from EN in the ‘before group’ vs 15 (12%) patients in the ‘after group,’ *p* = 0.002 (Additional file 5: Table S3). For these patients, enteral calories intakes were 624 (443) kcal versus 914 (615) kcal (*p* = 0.37) at day 3, and 685 (558) kcal versus 779 (581) kcal (*p* = 0.63) at day 5, for the ‘before’ and ‘after’ groups, respectively (Additional file 5: Table S3). During these first three days of ICU stay, 14 patients (11%) did not benefit from any EN, whereas 99 patients (77%) were treated with inefficient EN (enteral and/or oral quantities lower than 500 kcal), among them 87 (88%) had a justified medical reason for this enteral feeding failure. Of the 114 patients for whom an EN was initiated at ICU admission, enteral intolerance was diagnosed in 49 patients (43%). During the first three days of ICU stay, only 30 (22%) patients benefited from PN in the ‘before group’ versus 88 (69%) patients in the ‘after group’ (*p* < 0.0001). For these patients, PN calories intakes were 1217 (469) kcal versus 1488 (587) (*p* = 0.028) at day 3, and 1306 (505) kcal versus 1444 (560) kcal (*p* = 0.18) at day 5, for the ‘before’ and ‘after’ groups, respectively (Additional file 5: Table S3). During the first 5 days of ICU admission, the number of patients who did not receive neither PN nor EN was significantly lower in the ‘after group’ as compared with the ‘before group’ (*p* < 0.0001) (Additional file 5: Table S3). During the ICU stay, cumulative caloric deficit significantly decreased in the ‘after group’ as compared with the ‘before group’ (Additional file 1: Table S1). In addition, the number of underfed patients (daily deficit greater than 500 kcal) was significantly more important in the ‘before group’ versus the ‘after group’: 119 (86%) patients versus 41 (32%) on day 3, and 76 (67%) versus 24 (20%) on day 5, respectively (Additional file 1: Table S1). At ICU discharge, weight loss was similar between the two groups (*p* = 0.53) (Table 1). Overfeeding (excess calories of the day > 500 kcal) was similar between the two groups, on day 3, eight patients (6.25%) were overfed in the ‘after group’ versus 3 (2.17%) (*p* = 0.12) in the ‘before group’, and 14 patients (11.67%) versus 5 (4.42%) (*p* = 0.06) on day 5 (Additional file 1: Table S1).

**Table 2:** Number of days reaching caloric and protein targets during ICU stay

	‘Before group’		‘After group’		Intensity rate ratio	*p*
	Number of days in ICU days within target	Intensity rate	Number of days in days within target	Intensity rate		
All patients	*n* = 147		*n* = 128			
Calories	*2.08 (4.29)*	*18.4*% *[14.4–23.5*%*]*	*4.13 (6.13)*	*28.8*% *[24.6–33.7*%*]*	*1.57 [1.17–2.10]*	*0.0025*
Proteins	*0.83 (3.09)*	*7.3*% *[4.3–12.5*%*]*	*4.09 (5.55)*	*28.3*% *[24.4–32.8*%*]*	*3.86 [2.21–6.73]*	< *0.0001*
Subgroup analysis						
Neutropenic	*n* = 43		*n* = 57			
Calories	2.28 (4.04)	23.4% [15.2–36.0%]	5.33 (6.53)	33.7% [27.4–41.5%]	1.44 [0.89–2.33]	0.13
Proteins	0.86 (3.14)	8.8% [3.1–24.8%]	4.61 (5.29)	29.2% [24.4–35.0%]	3.31 [1.16–9.43]	0.025
Severe patients	*n* = 41		*n* = 40			
Calories	2.00 (3.26)	20.9% [13.6–32.0%]	3.75 (4.01)	26.1% [20.2–33.8%]	1.25 [0.76–2.07]	0.38
Proteins	*0.49 (1.50)*	*5.1*% *[2.0–12.6*%*]*	*3.41 (4.75)*	*23.3*% *[18.3–29.8*%*]*	*4.59 [1.79–11.76]*	*0.0015*
Allogeneic HSCT	*n* = 32		*n* = 38			
Calories	2.56 (3.72)	23.6% [15.5–35.8%]	4.42 (4.62)	29.8% [23.8–37.4%]	1.27 [0.79–2.04]	0.33
Proteins	1.47 (4.65)	13.5% [5.2–34.9%]	4.34 (5.00)	29.3% [22.4–38.4%]	2.17 [0.81–5.82]	0.12

Significant results are represented in italic after Bonferroni correction

*HSCT* hematopoietic stem cell transplantation

**Fig. 1:**
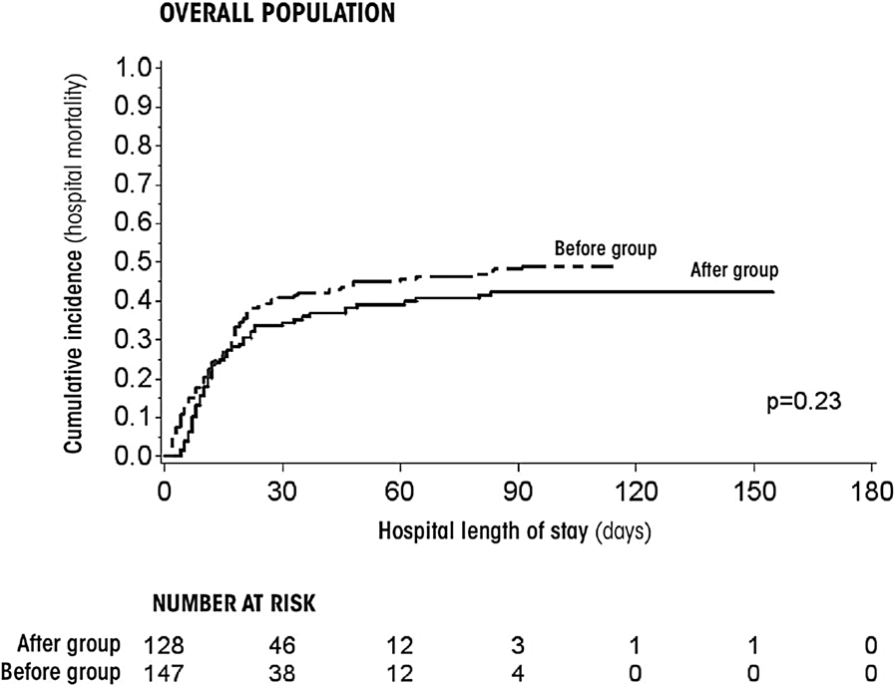
Hospital survival according to the use of CDSS (after group) for overall population

Regarding protein delivery (Table 2), during the ICU stay, the rate of days in compliance with protein target relatively increased in the ‘after group’ by more than 50% as compared with the ‘before group’ (7.3% [4.3–12.5%] vs. 28.3% [24.4–32.8%]; IRR = 3.86 [2.21–6.73], *p* < 0.0001). By considering Bonferroni correction, this trend was also significant in severe patients (Table 2). Days with IMV, vasopressors treatment, RRT, antibiotics, ICU-acquired infection, and organ failures regardless of neutropenia were similar between the two groups of patients (Table 3). Hospital death cumulative incidences were similar for the two nutritional strategies in overall population (Fig. 1) and in the subgroup of allogeneic HSCT. Conversely, hospital survival was significantly higher in the ‘after group’ compared to the ‘before group’ for neutropenic (*p* = 0.018), and severe patients (*p* = 0.023). Interestingly, SAPSII score was similar at ICU admission between ‘before group’ and ‘after group’ for neutropenic patients (53.00 [43.00–63.00] vs. 56.00 [46.00–65.00], *p* = 0.45, respectively) and severe patients (68.00 [61.00–75.00] vs. 64.50 [60.00–71.50], *p* = 0.18, respectively).

**Table 3:** Outcomes during ICU stay

	‘Before group’ (*n* = 147)		‘After group’ (*n* = 128)		Intensity rate ratio [CI 95%]	*p*
	Intensity rate		Intensity rate			
Support						
Days with IMV	3.99 (7.45)	35.2% [28.6–43.3%]	4.83 (8.89)	33.7% [27.0–42.1%]	0.96 [0.71–1.30]	0.79
Days with vasopressors	2.76 (4.16)	24.3% [18.9–31.3%]	2.56 (3.35)	17.9% [14.5–22.1%]	0.74 [0.53–1.02]	0.07
Days with dialysis	1.06 (2.67)	9.4% [6.2–14.2%]	2.06 (4.72)	14.4% [10.3–20.2%]	1.54 [0.90–2.63]	0.12
Days with antibiotics	8.90 (7.85)	78.3% [69.4–88.3%]	12.38 (11.34)	86.4% [81.9–91.2%]	1.10 [0.97–1.26]	0.14
Number of antibiotics used during ICU stay	3.07 (1.86)		3.52 (2.03)		0.91 [0.73–1.12]	0.37
Infections						
ICU-acquired infection	34 (23.13)		40 (31.25)			0.13
VAP	18 (12.24)		16 (12.50)			0.95
Bacteremia	12 (8.16)		18 (14.06)			0.12
CVC-related infection	4 (2.72)		4 (3.13)			1
Urinary tract infection	5 (3.40)		8 (6.25)			0.27

*IMV* invasive mechanical ventilation, *ICI* intensive care unit, *VAP* ventilation associated pneumonia, *CVC* central venous catheter

## Discussion

We report herein, in this before/after study, on 275 critically ill hematology patients admitted to the ICU over 5-year period. Energy and protein intakes were delivered using standard protocol in the 147 patients (53%) of the ‘before group’ and using a CDSS in order to reach every day predefined caloric and protein targets accordingly to the catabolic or anabolic status in the 128 patients (47%) of the ‘after group.’ We showed that the use of CDSS allows to relatively increase the rate of days in compliance with caloric and protein targets in the ‘after group’ by more than 50% as compared with the ‘before group.’ Interestingly, compliance rates were low and only reached 30% after intervention. Hospital mortality, ICU-acquired infection, and hospital and ICU length of stay were similar in the two groups of patients. Exploratory analysis showed that hospital mortality was lower in the ‘after group’ for neutropenic and severely ill patients.

**Fig. 2:**
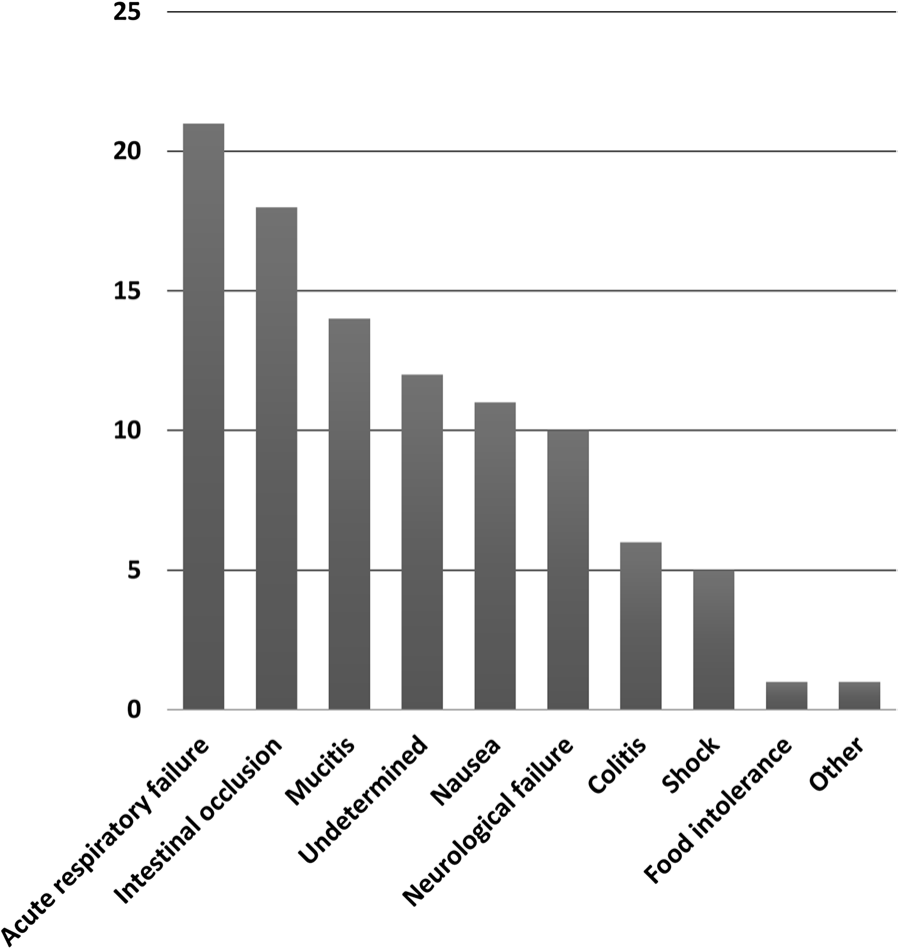
Reasons for oral and enteral nutrition failure in the ‘after group.’ *ARF* acute respiratory failure

In critically ill patients, guidelines recommend the use of early EN during the acute phase of the disease in order to prevent muscle wasting, infections, delayed recovery, and mortality [7, 8]. However, early EN may induce vomiting and gut ischemia during septic shock [21]. In hematology patients and in allogeneic HSCT recipients, guidelines recommend the use of early EN since it seems to be associated with better survival, less acute graft-versus-host disease (GVHD), and faster neutrophil recovery as compared with PN [11, 12, 22]. One striking finding from our study is that EN was difficult to realize in critically ill hematology patients, in fact the rate of EN days during ICU stay was low and about 13% and 25% for the before and after groups, respectively (Additional file 2: Table S2). The use of CDSS was however significantly helpful in this setting, particularly in the first 5 days of ICU admission during which only 4% of patients were nourished using EN before the use of CDSS and 26% after (Additional file 3: Table S3). We showed that the causes of EN failure could be frequently related to specific hematologic clinical patterns such as colitis or mucositis (Fig. 2). For these reasons, PN seemed to be the more appropriate feeding route as PN represented about 50% (‘before group’) of nutrition intakes during ICU stay and significantly increased to 63% (Additional file 2: Table S2). Accordingly, during the first 5 days of ICU admission, PN was the most frequent feeding route and the rate of patients nourished with PN significantly increased to 76% after CDSS use (Additional file 3: Table S3). Early nutrition appeared to be a crucial appointment in our study since 45% (‘after group’) and 58% (‘before group’) of the total caloric deficit was reached during the first 5 days of ICU admission (Additional file 1: Table S1). Thus, during the same period and after CDSS use, the number of patients who benefited from early nutrition (PN and/or EN) significantly increased as well as caloric intake (Additional file 3: Table S3). CDDS seemed to be an interesting tool to optimize energy intake and limit energy deficit in this setting [10, 23–25]. Our results are in line with recent randomized clinical trials in which early PN was safe and as effective as enteral route which was associated with gastrointestinal complications [21, 26]. In NUTRIREA-2 study [21], caloric target was designed to match the course of acute disease in two steps (the acute and the recovery phases) [27], we used similar design for energy delivery. Despite this strategy, our patients were underfed due to the low compliance rate regarding energy target only reaching the value of 30% even after CDSS use. Similar results were found in the CALORIES study [26] in which the percentage of patients in whom energy target was met was about 30% for each day of the study period. Taken together, these results underline that energy intake might be better evaluated with daily rather than global assessment in which underfeeding might be underestimated. Indeed, in our study, the mean daily calorie intake in the ‘after group’ was comparable to recent data [28], about 1200 kcal/day (Additional file 1: Table S1), suggesting a moderate underfeeding contrasting with a more profound underfeeding when the assessment was detailed every day. Importantly, the low rate of compliance regarding caloric target was also due to overfeeding situations, thus 12% of patients were overfed at day 5 in the ‘after group’ (Additional file 1: Table S1). Finally, the failure to reach caloric target might be overestimated since the number of days in compliance with calories target was defined as days on which caloric delivery fitted the targets ± 15%. This definition appears to be very strict as compared with recent studies in which underfeeding was defined as caloric intake below 70% of the target [29].

There is growing evidence that protein intake might also affect outcomes [30, 31]; however, the best protein intake in critically ill patients is still unknown. Recent recommendations on protein intake for critically ill patients are only based on studies with a low level of evidence [32]. In the present study, we showed that the use of CDSS was associated with both a significant increase in protein delivery and decrease in protein deficit during ICU stay. Thus, mean daily protein intake after intervention was about 56 g/day (Additional file 1: Table S1) and similar to that recently described by Nicolo et al. [28]. As for energy intake, 47% (‘after group’) and 52% (‘before group’) of the total protein deficit was reached during the first 5 days of ICU admission (Additional file 1: Table S1) underlining once again the potent crucial role of early PN for those patients. Intensivists usually focus on caloric rather than protein goals, and the parenteral nutrition products we used have compositions that could not provide 1.5 g of protein/kg/day without overfeeding. Thus, prescribers needed to tradeoff between high calories or high proteins. Glutamine, added to total PN, could be a key to higher protein delivery without a caloric overshoot [33]. During the ‘after period,’ glutamine was available in our institution and thus integrated in the CDSS and could have allowed us to better reach our protein targets.

The hospital mortality was about 46% consistent with recent results [1]. For the overall population, CDSS use did not translate into a mortality benefit. Because mortality was a secondary objective, our sample was not powered to show significant mortality difference. However, exploratory analysis showed a significant effect on hospital outcome after CDSS use for the neutropenic and severe patients but not for HSCT recipients. Interestingly, SAPSII score was similar at ICU admission between ‘before group’ and ‘after group’ for neutropenic and severe patients. These results should be interpreted cautiously nevertheless they reinforce the hypothesis of a beneficial effect on outcome of using CDSS. After intervention, we described in severe patients, a significant increase in days within protein target, while this effect was not retrieved for calories target. Taken together, these results suggest that protein intake in critically ill hematology patients might be a crucial step of ICU management. Indeed, it has been recently shown in critically ill patients that if the basal amount of protein is provided, restricted calories intake did not impact on outcome [29]. Recent data have also shown in patients who remained in the ICU ≥ 4 days and achieved at least 80% of prescribed protein intake a lower mortality than those achieving < 80% of prescribed. In this situation, percentage of prescribed energy intake was not associated with outcomes [28]. Whether reciprocal relationships exist between calorie and protein intakes exist in severe hematology patients remains to be determined.

Our study presents several limitations. First, the distinction between the acute phase and the recovery period was based on the clinician’s choice. Because there is no consensus on the definitions of acute phase and recovery phase, the retrospective analysis of the clinician’s evaluation might have led to significant variability from one patient to another. Such variability might have influenced the results. Second, due to the retrospective nature of the study, we were unable to collect the causes of non-use of enteral nutrition among the 14 patients who did not benefit. However, the causes of failure of enteral nutrition are detailed Fig. 2. Third, we did not plan to collect cases of refeeding syndrome; however, in our daily practice. these cases are rare, whereas the ICU admission of severely malnourished patients was relatively frequent (22%).

In conclusion, we showed that the use of CDSS allows to increase relatively the rate of days in compliance with caloric and protein targets by more than 50%. Interestingly, compliance rates were low and only reached 30% after intervention. Hospital mortality, ICU-acquired infection and lengths of stay were similar in the two groups.

## Additional files

**Additional file 1.** Implementation of the nutrition protocols during the 2 periods.

**Additional file 2.** Description of feeding products during the study period.

**Additional file 3: Table S1.** Intakes and cumulative deficit for calories and proteins during ICU stay.

**Additional file 4: Table S2.** Type of nutrition during ICU stay.

**Additional file 5: Table S3.** Route and caloric intake during the five first days of ICU admission.

## Data Availability

The original datasets used and analyzed during the current study are available from the corresponding author on reasonable request.
