# Supplementary material for: Impact of a computer-assisted decision support system (CDSS) on nutrition management in critically ill hematology patients: the NUTCHOCO study (nutritional care in hematology oncologic patients and critical outcome)
Source: Ann Intensive Care. 2019 May 7;9:53. doi: 10.1186/s13613-019-0527-6 (PMC6505002; doi:10.1186/s13613-019-0527-6)
Supplement: Supplementary file 1 — Additional file 1. Implementation of the nutrition protocols during the 2 periods. [file 13613_2019_527_MOESM1_ESM.docx]

**Additional file 1**

During the ‘before period’, paper-based guidelines for nutritional support were available to every ICU physician. Guidelines incorporated international guidelines for nutritional support regarding haematology patients and critically ill patients.^9;14-16^ During this period and in accordance with nutritional guidelines, nutritional prescription was let at the discretion of the attendant intensivist. Following the ‘before period’, a CDSS was designed as a tool to provide clinicians with essential guideline-based information concerning nutritional support in the ICU and haematology patients.^9;14-16^ This CDSS has been locally designed by Dr. Chow-Chine and has been added as a nutrition prescription module to our prescription software in our ICU information system (MetaVision®, iMDsoft, Tel Aviv, Israel). The variables needed to activate this module were age, sex, weight, BMI, acute phase or recovering period and nutritional status. The CDSS could be accessed from every computer in the ICU, including all computers used to document electronic patient files. The CDSS contained pathways to prescribe nutrition adapted to the critically ill haematology patient’s state, mainly based on the ESPEN guidelines.^9;14-16^ This nutritional computerized tool was added to our existing ICU software. This CDSS let the intensivist simulate, through a combination of routes and products, the closest-to-target caloric and protein prescription for the day, and generate a prescription accordingly. The CDSS monitored a comprehensive hourly, daily, and cumulative caloric intake count and protein intake count, and calculated the caloric balance defined as the difference between daily caloric intake and target, and protein balance likewise. All daily balances were checked for coherence on exit by one of the investigators (FE and AH). All sources of calories and protein (parenteral, enteral, drugs and intravenous fluids and drips) were automatically computed by the CDSS. Both prescribed and delivered calories and proteins were therefore available on an energy prescription and energy balance screen sheet, throughout the whole stay. This protocol implicated the whole ICU team: physicians, nurses, dieticians, after a period of implementation of six months without any inclusions. Prescribers were the same intensivists throughout the study period.
