# Supplementary material for: Impact of a computer-assisted decision support system (CDSS) on nutrition management in critically ill hematology patients: the NUTCHOCO study (nutritional care in hematology oncologic patients and critical outcome)
Source: Ann Intensive Care. 2019 May 7;9:53. doi: 10.1186/s13613-019-0527-6 (PMC6505002; doi:10.1186/s13613-019-0527-6)
Supplement: Supplementary file 2 — Additional file 2. Description of feeding products during the study period. [file 13613_2019_527_MOESM2_ESM.docx]

Feeding products (Additional file 2)

Different enteral feeding solutions were used during the two periods. During the ‘before period’ they included standard or high energy and/or relatively high protein enteral solution (Sondalis ISO®, Sondalis HP®, Sondalis HP fibres®, Nestlé). During the ‘after period’, they included other standard or high energy and/or relatively high protein enteral solution (Realdiet standard Fibres ®, Realdiet HP Fibres®, Realdiet HC®, DHN), in addition enteral omega-3 enriched products were also introduced (Megareal®, Fresenius Kabi). Regarding PN nutrition, two brands of industrial PN 3-chamber bag solutions using standard or high energy (with or without omega-3 enriched products) and/or relatively high protein parenteral solution were delivered (‘before period’: Kabiven 800®, Kabiven 1200®, Kabiven 1600®, Fresenius Kabi; Oliclinomel N7-1000®, Oliclinomel N8-800®, Baxter; ‘after period’: Kabiven 1600®, Smofkabiven®, Fresenius Kabi). Intravenous glutamine (i.e., 110 g, including 33 g L-alanyl-L-glutamine as Dipeptiven® Fresenius Kabi) was also used during the ‘after period’, for patients in the acute phase of critical illness and on total TPN, or near total parenteral nutrition (NTPN) defined as enteral feed<500 kcal/day.
