# Supplementary material for: Impact of a computer-assisted decision support system (CDSS) on nutrition management in critically ill hematology patients: the NUTCHOCO study (nutritional care in hematology oncologic patients and critical outcome)
Source: Ann Intensive Care. 2019 May 7;9:53. doi: 10.1186/s13613-019-0527-6 (PMC6505002; doi:10.1186/s13613-019-0527-6)
Supplement: Supplementary file 3 — Additional file 3: Table S1. Intakes and cumulative deficit for calories and proteins during ICU stay. [file 13613_2019_527_MOESM3_ESM.docx]

**Table S1: Intakes and cumulative deficit for calories and proteins during ICU stay**

|  | Before (n=147) | After (n=128) | P value |
| --- | --- | --- | --- |
| **Calories intake** |  |  |  |
| Daily calories intake(kcal/d) | 647 (499) | 1152 (513) | <0.0001 |
| Total calories intake (kcal/kg) | 148 (259) | 263 (293) | <0.0001 |
| Daily calorie intake (kcal/kg/d) | 9.38 (7) | 16.43 (7) | <0.0001 |
| **Protein intake** |  |  |  |
| Daily protein intake (g/d) | 20 (24) | 56 (35) | <0.0001 |
| Total protein intake(g/kg) | 5 (11) | 13 (15) | <0.0001 |
| Daily protein intake (g/kg/d) | 0.29 (0.35) | 0.79 (0.49) | <0.0001 |
| **Cumulative caloric deficit (kcal**) |  |  |  |
| At day 3 | 3172 (1479) | 1996 (1445) | <0.001 |
| At day 5 | 5066 (2781) | 2629 (2676) | <0.001 |
| At ICU discharge | 8722 (12361) | 5786 (7304) | <0.001 |
| **Cumulative protein deficit (g**) |  |  |  |
| At day 3 | 236 (81) | 159 (109) | <0.001 |
| At day 5 | 400 (154) | 227 (188) | <0.001 |
| At ICU discharge | 763 (1059) | 487 (564) | 0.0022 |
| **Patients with cumulative caloric deficit < 500 kcal (underfeeding)** |  |  |  |
| At day 3 | 119 (86) | 41 (32) | <0.001 |
| At day 5 | 76 (67) | 24 (20) | <0.001 |
| **Patients with overfeeding > 500kcal** |  |  |  |
| At day 3 | 3 (2) | 8 (6) | 0.12 |
| At day 5 | 5 (4) | 14 (12) | 0.055 |

ICU: intensive care unit
