# Supplementary material for: Impact of a computer-assisted decision support system (CDSS) on nutrition management in critically ill hematology patients: the NUTCHOCO study (nutritional care in hematology oncologic patients and critical outcome)
Source: Ann Intensive Care. 2019 May 7;9:53. doi: 10.1186/s13613-019-0527-6 (PMC6505002; doi:10.1186/s13613-019-0527-6)
Supplement: Supplementary file 4 — Additional file 4: Table S2. Type of nutrition during ICU stay. [file 13613_2019_527_MOESM4_ESM.docx]

**Table S2: Type of nutrition during ICU stay**

|  | Before (n=147) |  | After (n=128) |  | Intensity Rate Ratio | P |
| --- | --- | --- | --- | --- | --- | --- |
|  | Number of days in ICU | Intensity Rate | Number of days in ICU | Intensity Rate |  |  |
| With EN | 1.51 (6.89) | 13.3% [7.4%-24.0%] | 3.63 (8.33) | 25.3% [18.9%-33.8%] | 1.90 [0.98-3.66] | 0.056 |
| With exclusive EN | 0.07 (0.76) | 0.7% [0.1%-3.3%] | 0.06 (0.51) | 0.4% [0.1%-1.7%] | 0.66 [0.08-5.52] | 0.70 |
| **With PEN** | **5.58 (9.35)** | **49.2% [43.0%-56.3%]** | **9.09 (9.18)** | **63.4% [58.0%-69.4%]** | **1.29 [1.10-1.52]** | **0.0021** |
| **With exclusive PEN** | **3.46 (5.74)** | **30.5% [25.7%-36.1%]** | **0.60 (1.74)** | **4.2% [2.6%-6.8%]** | **0.14 [0.08-0.23]** | **<0.0001** |
| With Combined (PEN+EN) | 0.82 (3.37) | 7.3% [4.2%-12.5%] | 1.61 (3.33) | 11.2% [8.8%-15.3%] | 1.55 [0.83-2.89] | 0.17 |

EN: enteral nutrition; PEN: Parenteral nutrition
