# Supplementary material for: Impact of a computer-assisted decision support system (CDSS) on nutrition management in critically ill hematology patients: the NUTCHOCO study (nutritional care in hematology oncologic patients and critical outcome)
Source: Ann Intensive Care. 2019 May 7;9:53. doi: 10.1186/s13613-019-0527-6 (PMC6505002; doi:10.1186/s13613-019-0527-6)
Supplement: Supplementary file 5 — Additional file 5: Table S3. Route and caloric intake during the five first days of ICU admission. [file 13613_2019_527_MOESM5_ESM.docx]

**Table S3: Route and caloric intake during the five first days of ICU admission**

| Characteristics | ‘Before group’  Day 3, n= 138  Day 5 , n= 113 | ‘After group’  Day 3, n= 128  Day 5 , n= 120 | p-value |
| --- | --- | --- | --- |
| **Enteral nutrition** |  |  |  |
| Day 3 (nb of patients) | 3 (2) | 15 (12) | 0.002 |
| Day 3 (kcal) | 624 (543) | 914 (615) | 0.37 |
| Day 5 (nb of patients) | 5 (4) | 31 (26) | <0.0001 |
| Day 5 (kcal) | 685 (558) | 779 (581) | 0.63 |
| **Parenteral nutrition** |  |  |  |
| Day 3 (nb of patients) | 30 (22) | 88 (69) | <0.0001 |
| Day 3 (kcal) | 1217 (469) | 1488 (587) | 0.028 |
| Day 5 (nb of patients) | 52 (46) | 91 (76) | <0.0001 |
| Day 5 (kcal) | 1306 (505) | 1444 (560) | 0.18 |
| **Enteral and/or parenteral nutrition** |  |  |  |
| Day 3 (nb of patients) | 31 (22) | 93 (73) | <0.0001 |
| Day 3 (kcal) | 1238 (503) | 1556 (603) | 0.015 |
| Day 5 (nb of patients) | 52 (46) | 97 (81) | <0.0001 |
| Day 5 (kcal) | 1372 (529) | 1604 (679) | 0.061 |
| **Combined nutrition (EN+PEN)** |  |  |  |
| Day 3 (nb of patients) | 2 (1) | 10 (8) | 0.012 |
| Day 3 (kcal) | 1472 (959) | 1904 (701) | 0.52 |
| Day 5 (nb of patients) | 5 (4) | 25 (21) | 0.0002 |
| Day 5 (kcal) | 1815 (658) | 2113(707) | 0.43 |
| **Neither enteral nor parenteral nutrition** |  |  |  |
| Day 3 (nb of patients) | 104 (75) | 35 (27) | <0.0001 |
| Day 3 (kcal) | 404 (388) | 872 (679) | <0.0001 |
| Day 5 (nb of patients) | 59 (52) | 23 (19) | <0.0001 |
| Day 5 (kcal) | 382 (307) | 1065 (724) | <0.0001 |

EN: Enteral nutrition; PEN: Parenteral nutrition
